# Safety and efficacy of flowable microfibrillar collagen hemostat in an ovine model of lumbar laminectomy and open durotomy compared with the gelatin-thrombin hemostatic matrix and control

**DOI:** 10.3389/fsurg.2026.1760993

**Published:** 2026-06-09

**Authors:** Amie Rossi, Kasia Bradbury, Kingsley Abode-Iyamah, Angela Bohnen, Darcy Gagne

**Affiliations:** 1 Becton, Dickinson and Company, Warwick, RI, United States; 2 Department of Neurosurgery, Mayo Clinic, Jacksonville, FL, United States; 3 Neurosurgery One, Littleton, CO, United States

**Keywords:** collagen, flowable hemostat, gelatin, laminectomy, open durotomy, spinal cord, spinal surgery, thrombin

## Abstract

**Background:**

Effective hemostasis in spinal surgery is critical, especially near sensitive structures such as the spinal cord and dorsal root ganglia. Flowable hemostatic agents offer targeted control in irregular, hard-to-reach areas. This study evaluates the safety and efficacy of flowable collagen hemostat (FC) in an ovine lumbar laminectomy model with open durotomy by making a direct comparison with the gelatin hemostatic matrix with thrombin (GM) and a no-treatment control (Sham).

**Methods:**

Fifty-four Polypay sheep (27 F, 27 M) underwent lumbar laminectomy and open durotomy and then received FC, GM, or no treatment (*n* = 18/group). Hemostasis was assessed at 3 min postapplication without tamponade. Neurological examinations were performed at periodic intervals, and tissues were collected on days 7, 45, and 120 for a histological analysis.

**Results:**

Hemostasis was achieved in 100% of FC-treated sites, 94.4% of GM, and 72.2% of Sham. Sham-treated animals exhibited more severe and prolonged neurological deficits compared with those treated with FC or GM. Inflammation decreased significantly across all groups, from mild/moderate on day 7 to minimal on day 45, and was barely detectable by day 120. FC was associated with favorable histological responses in both intradural and extradural spinal tissues, comparable to GM and Sham.

**Conclusion:**

Flowable collagen hemostat was safe and effective when applied directly to the spinal cord and surrounding neurological tissues during laminectomy with open durotomy. Its performance was comparable to that of the gelatin hemostatic matrix with thrombin in terms of hemostatic efficacy and histological outcomes, with both agents demonstrating superior neurological recovery compared with the untreated Sham group.

## 1 Introduction

Effective surgical hemostasis is critical for minimizing intraoperative and postoperative complications in spine surgery. Excessive bleeding can obscure the surgical field, prolong operative time, and increase the risk of surgical complications and neurological injury (1, 2). Bleeding complication rates in spinal procedures range from 1.63% to 3.57%, depending on the procedure type, with approximately 24% of hemorrhagic events occurring in thoracic and lumbar spine surgeries (3, 4).

Over the last decade, the number of neurological procedures has increased significantly, highlighting the importance of effective hemostasis (2, 5, 6). Safe and efficient bleeding control improves surgical outcomes, including shorter recovery times, less days in the hospital, reduced hospital cost, and earlier return to work (1, 7).

Topical hemostatic agents support physiological clotting mechanisms by targeting various stages of the hemostatic cascade. These agents can be involved in primary hemostasis, promoting platelet aggregation and adhesion (oxidized regenerated cellulose, polysaccharide particles, microfibrillar collagen, silica-based particles). Thrombin-containing agents can directly activate the coagulation cascade in the secondary pathway, resulting in fibrin clot formation. Others can stimulate the intrinsic pathway to initiate thrombin and fibrin generation to stabilize the clot (oxidized regenerated cellulose, microfibrillar collagen). They can also act through mechanical tamponade, concentrating platelets and clotting factors (gelatin or polysaccharide-based agents) (8–11).

Topical hemostatic agents can be especially beneficial in spinal surgery, where using traditional techniques such as suturing, cautery, or manual compression can be detrimental to the neurological tissue or challenging due to the proximity of the neurological structure and complex anatomy. Flowable hemostatic agents conform to anatomical structures and effectively control localized bleeding, offering the precision and adaptability required around delicate neural tissue (8, 12).

In this study, the safety and hemostatic efficacy of flowable collagen hemostat (FC) are assessed in an ovine model of lumbar laminectomy with open durotomy by making a direct comparison with the gelatin hemostatic matrix with thrombin (GM) and a no-treatment Sham control to evaluate relative performance and neurological outcomes.

## 2 Methods

The protocol for this project was reviewed and approved by the Institutional Animal Care and Use Committee of CBSET, Inc. (Lexington, MA, USA; IACUC project # I00380) and was in compliance with the Food and Drug Administration GLPs, as set forth in Title 21 of the United States CFR, Part 58, and OECD Principles of GLP [ENV/MC/CHEM(98)17], Japanese Good Laboratory Practice Standards for Safety Studies on Drugs and Medical Devices (Ordinance Nos. 21 and 37, respectively, of the Pharmaceutical Affairs Bureau, MHLW, Japan).

### 2.1 Materials

This study was designed to evaluate FC—Avitene™ Flowable Collagen Hemostat (BD, Warwick, RI, USA) (13), a new form of Avitene^TM^ Microfibrillar Collagen Hemostat (MCH) (BD, Warwick, RI, USA), which has been used in its flour form for over 40 years (14, 15). Comparative groups included the GM—Floseal® Hemostatic Matrix (the flowable gelatin matrix with human-derived thrombin; Baxter International Inc., Deerfield, IL, USA) (16) and untreated control (Sham).

### 2.2 Study design/animal groups

Fifty-four sheep (27 F, 27 M; 27.1–48.1 kg) were included in the study and were divided into three groups (*n* = 18 per group), namely, Sham, FC, and GM groups. Each group was further divided across three points (days 7, 45, and 120), with six animals per group evaluated at each time point (*n* = 6 per group per time point). Eighteen animals in the Sham group received no hemostatic treatment following dorsal laminectomy and durotomy creation and were evaluated for hemostasis after the 3-min observation period. Any excessive bleeding was controlled with bone wax and/or cautery. The other two groups, with 18 animals each, had a hemostatic device, FC or GM, applied directly to the spine and spinal cord via open durotomy and subsequently evaluated for the hemostasis after 3 min of observation, precision of application at the treatment site, device swelling, and migration.

### 2.3 Surgical procedure

Animals underwent a dorsal lumbar laminectomy via posterior access over L1–L2 or L2–L3, followed by a durotomy, approximately 1 cm, to expose the spinal cord. Because of anatomical differences, these vertebral levels do not directly correspond to the same vertebral levels in humans. The hemostatic devices were then applied directly to the spinal cord via open durotomy. Applications were performed on the exposed spinal cord, at the bleeding edges of the bone, and laterally within the laminectomy site where dissection had occurred around facet joints/transverse processes (targeting both extradural and intradural anatomies). The durotomy was intentionally left open to simulate the worst-case clinical scenario in safety assessment, in which hemostatic material may migrate between the suture line, resulting in the hemostat making direct contact with the spinal cord.

Respiratory alkalosis (ETCO_2_ between 20 and 25 mmHg, when possible) was induced to shrink CNS structures and improve access. Before durotomy and device application, approximately 1.0 mL cerebrospinal fluid (CSF) was collected for determining specific gravity, differential, protein, glucose, cytologic interpretation, and culture.

FC or GM was then applied directly to the spinal cord via the open durotomy, on top of the spinal cord (extradural), the bleeding edges of the bone, and laterally where dissection had occurred around facet joints/transverse processes within the laminectomy site. The Sham group received no treatment following an open durotomy. Hemostatic efficacy was evaluated under mild to moderate bleeding conditions. Following the application of the hemostatic agent, the injury site was observed for a period of 3 min. If no visible bleeding was present at the end of this observation window, the injury was declared hemostatic. This assessment was reconfirmed prior to surgical closure. In cases where bleeding persisted beyond the 3-min window, hemostasis was considered a failure and gentle pressure was applied to the bony edges and surrounding soft tissue—avoiding direct pressure on the spinal cord—to assist in achieving hemostasis. These interventions were documented accordingly. The amount of bleeding seen and biological variation (within the intended use of mild/moderate bleeding) dictated the number of devices used. The surgeon determined this amount by ensuring that the target bleeding site was sufficiently covered with the hemostatic agent—the full volume of the reconstituted syringe of the hemostatic device was allowed to be applied and this volume was documented. Any excessive bone bleeding outside of the intended usage for mild to moderate bleeding was controlled with bone wax and/or electrocautery in the Sham-, GM-, and FC-treated animals. The devices were evaluated for ease of handling, accuracy in reaching the desired location, swelling, and migration. Any excess material of either FC or GM was removed in accordance with the device instruction for use by gentle suction. In the Sham group, no hemostatic treatment was applied; any additional bleeding control measures (e.g., use of bone wax) were documented. Treatment sites were photographed before and after material removal, and hemostasis was confirmed before closure.

### 2.4 Neurological examination

Neurological examinations were performed once before surgery as a baseline (day −3 to day 0), daily for the first 7 days, day 14 ± 1, and day 21 ± 1, and before necropsy. Animal health was monitored, including clinical observations, neurological examinations, body weights/condition scores, and clinical pathology, generally at predetermined, regular intervals.

### 2.5 Euthanasia and necropsy

Animals were euthanized at days 7 ± 1, 45 ± 2, and 120, with 18 animals per time point (6 animals per group), and a comprehensive necropsy was performed. Each animal was euthanized under deep anesthesia with 1 mL/4.5 kg euthasol or sodium pentobarbital and 2 mEg/kg potassium chloride administered intravenously, in accordance with accepted American Veterinary Medical Association (AVMA) guidelines (17) Necropsy was performed to evaluate tissue responses and hemostatic material degradation. Animal health was monitored throughout the study period.

### 2.6 Histomorphological evaluation

The treatment sites, proximal/distal spinal cord and vertebral segments, and representative tissues were collected and processed for a histomorphologic evaluation. Tissues were processed for paraffin embedding and stained with hematoxylin and eosin (H&E) and Luxol fast blue stain.

Spinal cord sections and dorsal root ganglia (DRG) were examined for axonal and neuronal degeneration along with any other abnormalities that may have been attributable to FC or GM, including but not limited to the inflammatory/fibrotic response, and the presence/absence of the hemostatic device material.

Semiquantitative scoring was performed by a single board-certified veterinary pathologist and applied consistently across all samples using predefined criteria. The pathologist was not blinded during a histopathological evaluation. This was intentional, and in accordance with best practices described by Bolon et al. (18), which indicate that informed (non-blinded) microscopic assessment in toxicity studies can improve sensitivity and diagnostic accuracy, outweighing theoretical reduction in bias from masked microscopic examination.

Each histological parameter—inflammation and inflammatory cells (lymphocytes, plasma cells, macrophages, multinucleated giant cells, mast cells), fibrosis and fibroplasia, axonal degeneration, osteogenesis and osteonecrosis, and device presence—was evaluated according to prespecified criteria. In general, minimal findings correspond to rare, low-density cell infiltrates with negligible tissue involvement, whereas marked findings reflect dense, diffused infiltrates associated with affected tissue architecture. Intermediate grades represent an increasing extent and/or density infiltrates and associated tissue involvement. All findings were graded according to a four-point semiquantitative scale:

0 = no response/not present,

1 = minimal/focal/barely detectable,

2 = mild/focal or rare multifocal/slightly detectable,

3 = moderate/multifocal to confluent/easily detectable,

4 = marked/diffuse/overwhelming presence.

### 2.7 Statistical analysis

Ordinal scores were statistically compared across the FC, GM, and Sham groups. Histological scores were reported as mean ± SEM (standard error of the mean), median, and percent incidence. Data analysis was performed using Microsoft Excel and GraphPad Prism®.

Categorical outcomes (hemostatic success vs. failure) were analyzed using Fisher's exact test. Select parameters were statistically evaluated using Kruskal–Wallis ANOVA, followed by Dunn's *post hoc* test (group comparison at each time point) or the Mann–Whitney rank-sum test (to compare each group across time points) with GraphPad Prism® statistical software. Corrections for multiple comparisons were not applied to the Mann–Whitney rank-sum test, as all comparisons were prespecified *a priori*.

A *p*-value ≤ 0.05 was considered statistically significant. Formal statistical comparisons were not performed between groups with identical distributions, as no meaningful differences were observed.

## 3 Results

**Figure 1:**
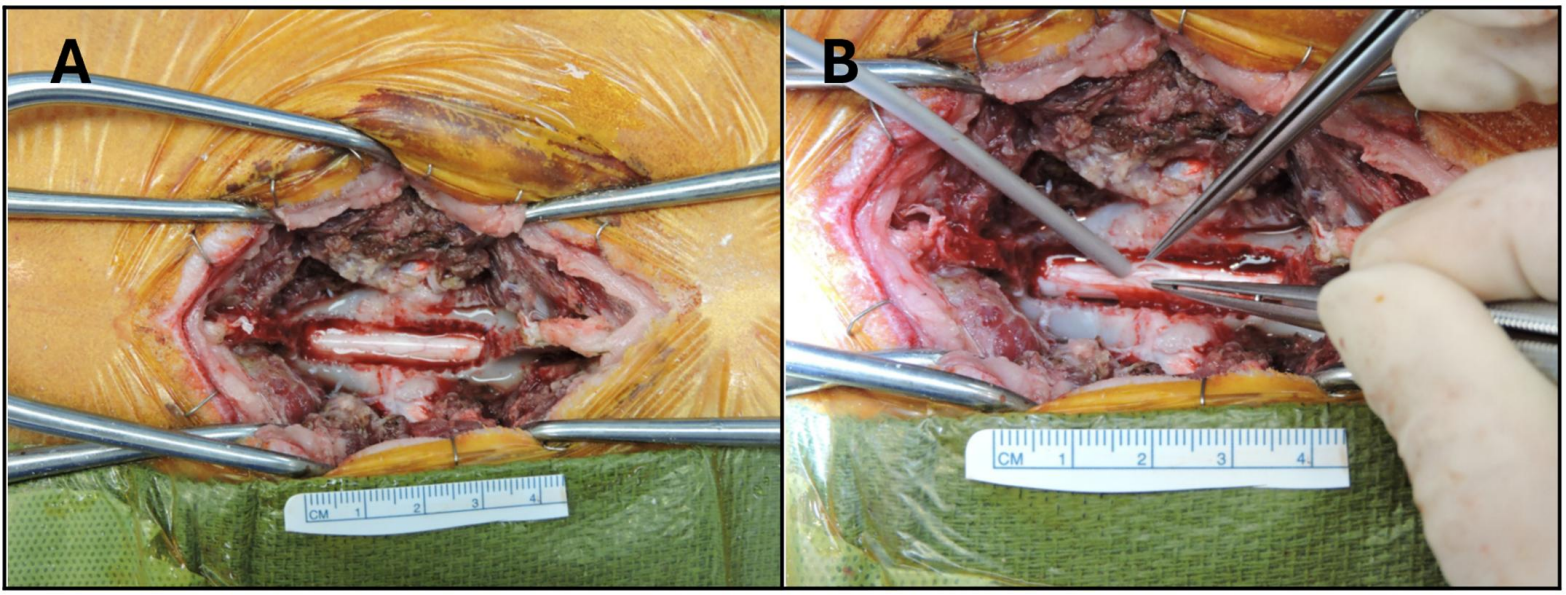
Laminectomy and durotomy site prior to CSF collection and hemostatic treatment. Representative images of laminectomy prior to CSF collection **(A)** and durotomy prior to treatment, held open with Rhotan's forceps **(B)** Image orientation with cranium to the left.

A lumbar laminectomy was performed on all animals. A clinically relevant volume of the device was used for treatment. Both hemostatic devices were applied directly to the spine and spinal cord via open durotomy. The volume of material applied for flowable collagen ranged from 3.4 to 4.0 mL (average of 3.9 mL), and for the gelatin matrix, it ranged from 3.0 to 5.0 mL (average of 3.8 mL). Excess material was removed from the treatment site of all animals in both groups after hemostasis was achieved. The durotomy was left open to ensure that the flowable hemostatic agents remained in contact with the spinal cord, allowing for the collection of safety data within the intradural compartment (direct contact with the spinal cord). The sites were reconfirmed to be hemostatic prior to closure. Representative images of laminectomy prior to CSF collection and durotomy prior to treatment are shown in Figure 1.

**Figure 2:**
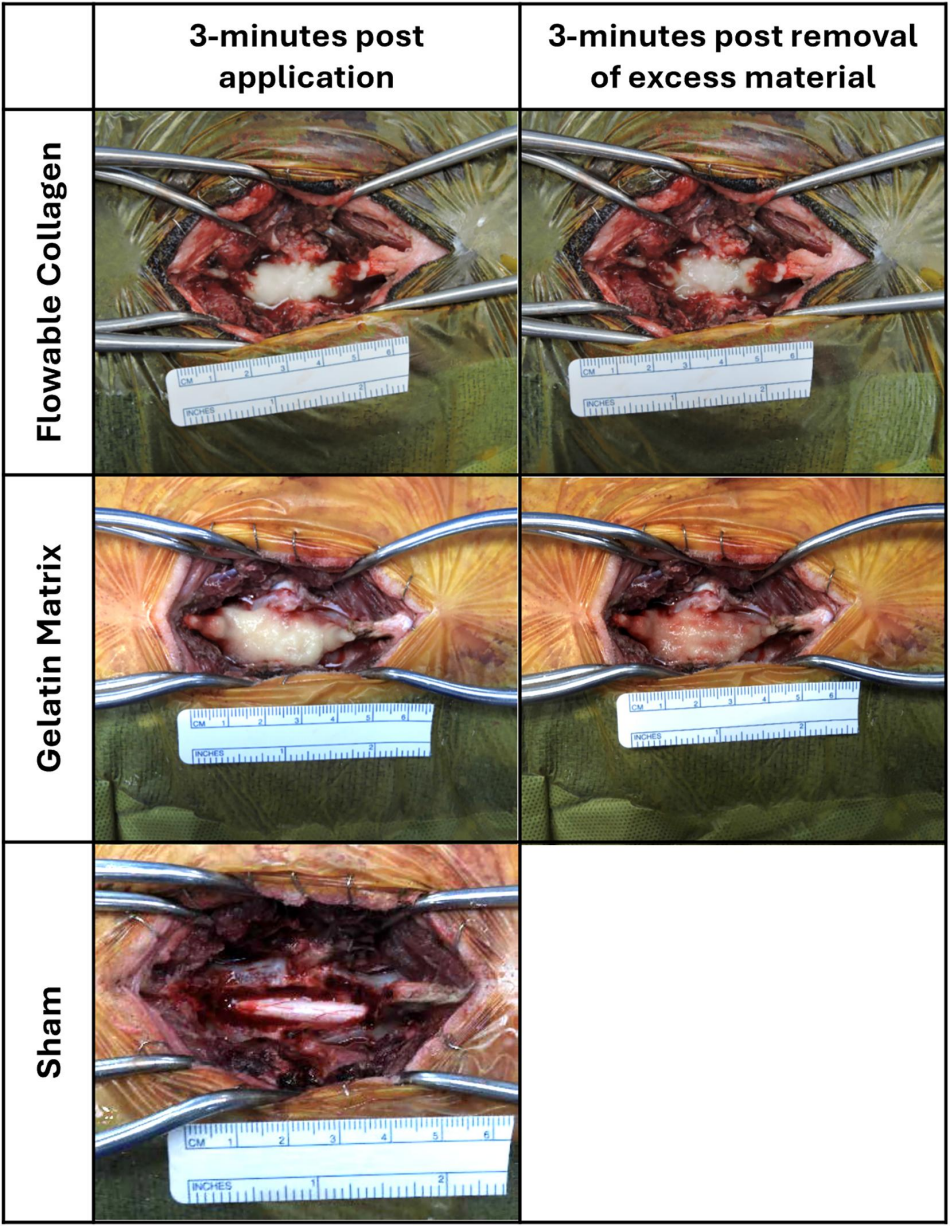
Hemostasis at the laminectomy site with FC, GM, and Sham. Representative images of hemostasis at the laminectomy site after the application of FC (top row), GM (middle row), and Sham control (bottom row). The images show a 3 min postapplication prior to the removal of excess material (left) and a 3 min postremoval of excess material (right). Image orientation with cranium to right.

**Figure 3:**
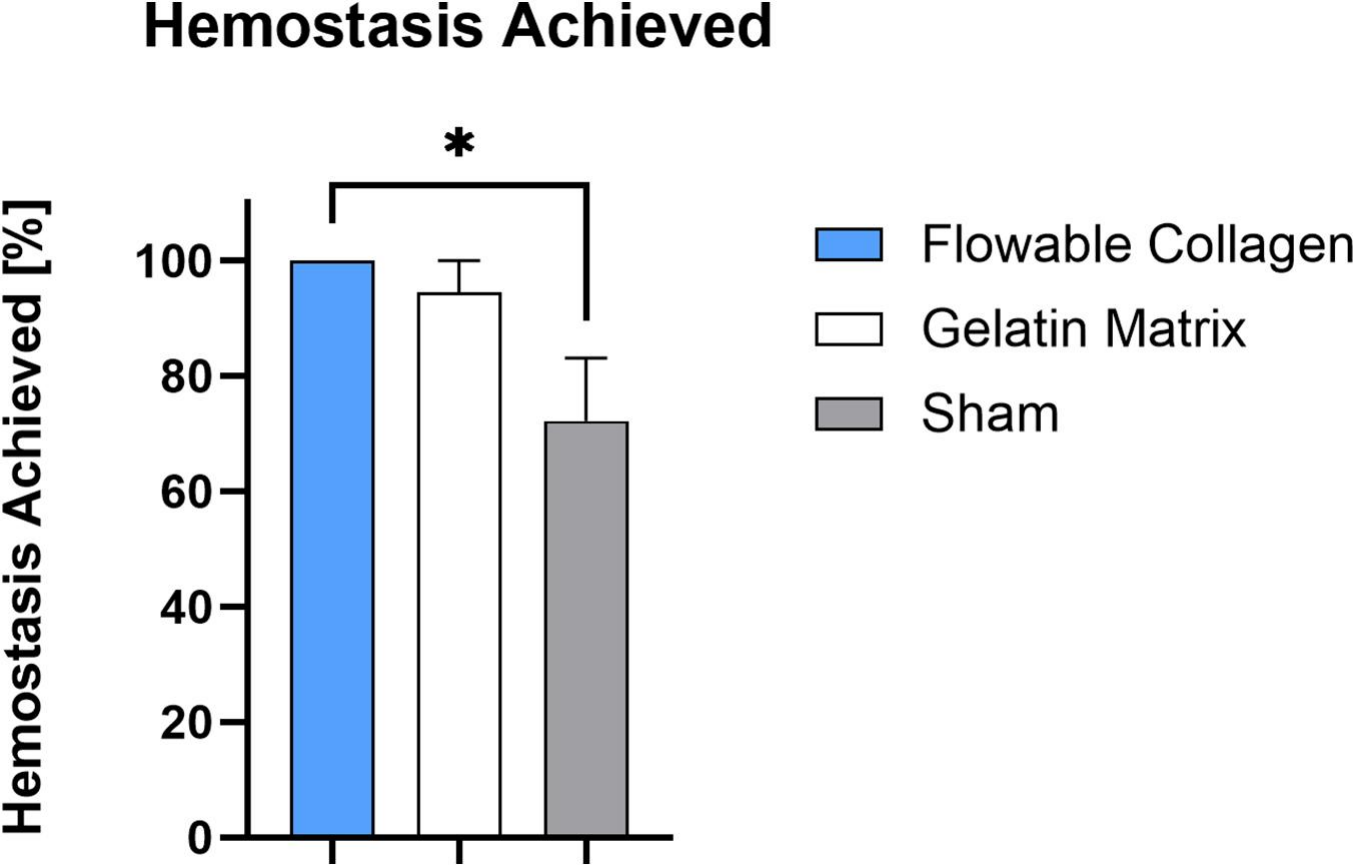
Hemostatic efficacy at 3 min for FC, GM, and Sham. The percentage of hemostasis achieved at 3 min for each treatment group (*n* = 6/group; mean ± SEM). *p < 0.05.

Both devices successfully targeted the injury site on day 0. No visible swelling of hemostatic device material or migration was observed, except after the third administration of GM in one animal. Because of the initial hemostasis failure, migration was noted. Hemostasis was confirmed prior to closure for all animals. Representative images of hemostasis at the laminectomy site are shown in Figure 2. Hemostasis was achieved at all treatment sites in the FC group (100%, 18/18). In comparison, 13 out of 18 (72.2%) sites in the Sham group and 17 out of 18 (94.4%) in the GM group were hemostatic after the 3-min observation period (Figure 3). Fisher’s exact test revealed a significant effect in the groups (*p* = 0.038), with a statistically significant difference identified between the FC and Sham group in the percentage of hemostasis achieved at 3 min (*p* = 0.046).

The proper placement of FC hemostatic material at the treatment site was assessed in a separate acute experiment. Details are provided in Supplementary Material 1.

No abnormalities were noted on the baseline neurological examinations, and no significant differences were found between groups in postsurgery examinations (*p* = 0.656). However, the Sham group reported the most adverse findings, with 7/18 animals showing prolonged pain and swelling at the incision site, which was resolved by day 7, except in one animal, which lasted for 25 days. Neurological deficits included the following: knuckling (unable to place feet/hooves/toes in plantigrade position), ataxia (swaying/instability during ambulation), and paresis (partial paralysis). In the FC group, 4/18 animals reported some adverse neurological findings, which were resolved by day 4. In the GM group, 5/18 showed adverse findings, which were resolved by day 14. An overview of the findings of the neurological examinations is presented in Table 1.

**Table 1:** Summary of neurological examination findings postsurgery.

Group	Animals with neurological findings	Nature of findings	Resolution
Flowable collagen	22% (4/18)	hunched posture ataxia toe touching drag in hind legs swelling due to bandage	Day 4
Gelatin matrix	28% (5/18)	hunched posture ataxia toe touching drag in the hind legs pain at the surgical site paralysis in the hind legs	Day 14
Sham	39% (7/18)	prolonged pain knuckling ataxia paresis hunched posture slowed responses drag in the hind legs paralysis in the hind legs swelling due to bandage	Prenecropsy (day 7) day 25

Four CSF samples were positive for *Staphylococcus* species. The presence of *Staph* was observed in one animal in each group on day 0 and in one animal in the Avitene Flowable group on day 7.

At necropsy, there was no reported macroscopic evidence indicative of major organ thromboembolism or impaired drainage/flow of the cerebrospinal fluid, and no macroscopic evidence of cerebral thromboembolism.

**Figure 4:**
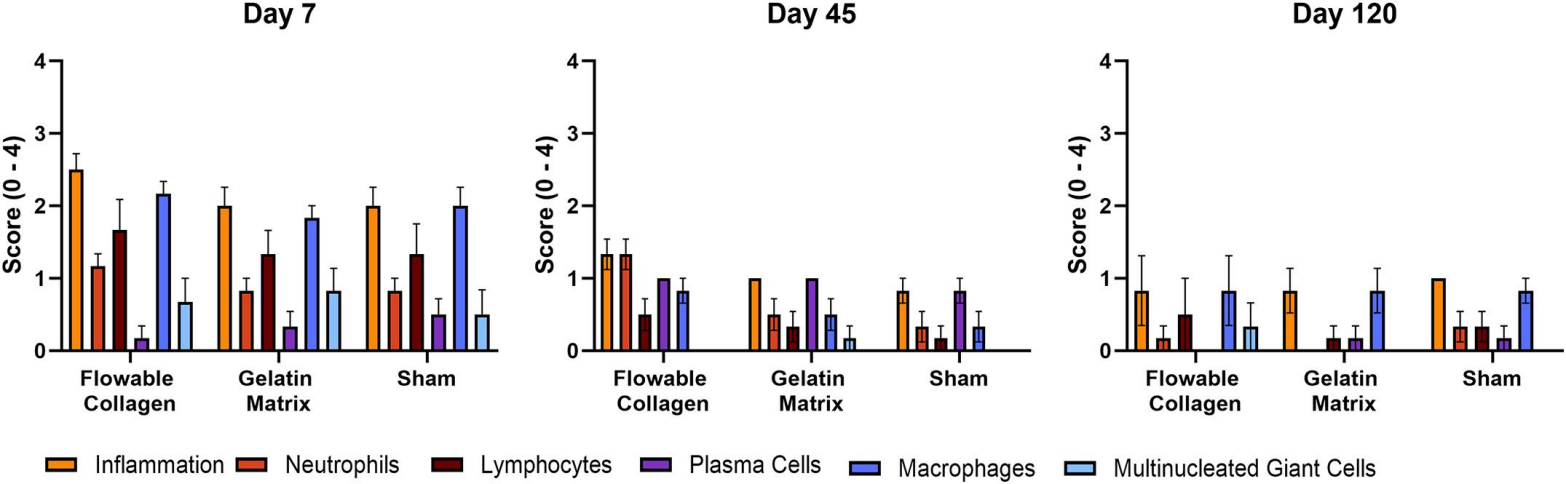
Inflammation scores over time post-treatment with FC, GM, and Sham. Semiquantitative histological scores showing inflammation and inflammatory cells at says 7, 45, and 120 (mean ± SEM; *n* = 6/group).

Inflammation and inflammatory cell scores decreased over time—from day 7 to day 120—in all groups, with some reductions reaching statistical significance. Specifically, the decrease in inflammation scores from day 7 to day 120 was statistically significant in all groups (*p* < 0.05). In the GM and Sham groups, the reduction in scores between day 45 and day 120 was also statistically significant (Supplementary Material 2, Supplementary Table S1). Scores for most inflammatory cells dropped over time, except for Avitene™ Flowable. Lymphocytes scores did not change between day 7 and day 45, and scores for multinucleated giant cells increased slightly from day 7 to day 45. These changes were not statistically significant. These findings are visually summarized in Figure 4.

**Figure 5:**
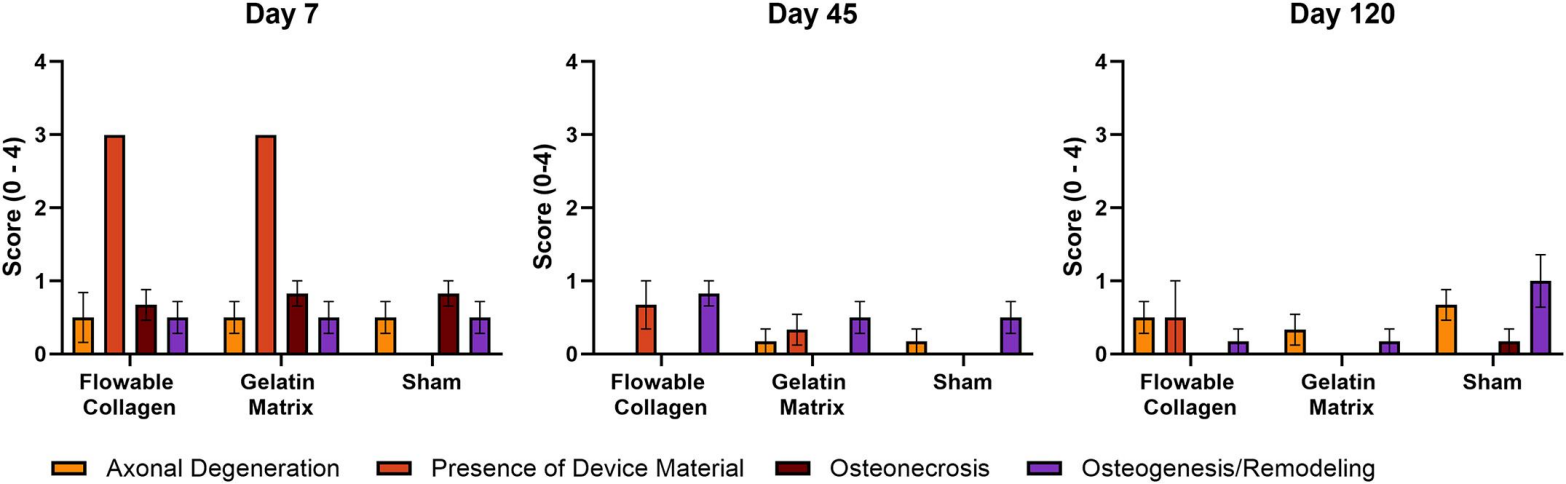
General histological observations at days 7, 45, and 120 post-treatment. Semiquantitative histological scores of general tissue observations at days 7, 45, and 120 (mean ± SEM; *n* = 6/group).

Both hemostatic devices were present in moderate quantities at the surgical site on day 7 and decreased significantly by day 45 (statistically significant *p* < 0.05) (Supplementary Material 2, Supplementary Table S2). FC was completely resorbed in the majority of 120 day animals, with only one out of six animals containing rare FC presence at day 120. GM was not observed on day 120 (Figure 5; Supplementary Material 2, Supplementary Table S2). Device remnants were primarily located within the connective tissue adjacent to the DRG. GM showed similar DRG findings, as well as rare quantities of device along the spinal cord dura.

Axonal degeneration was rare in all groups on day 7 and decreased further by day 45, with none observed in the FC group. On day 120, axonal degeneration was minimal in all three groups (Figure 5; Supplementary Material 2, Supplementary Table S2). Neuronal degeneration of the spinal cord or dorsal root ganglia was not observed.

Bone changes were minimal, and consistent with normal healing. Osteogenesis/remodeling was rare at the laminectomy site, with no significant differences among the groups or time points. Hemorrhage from surgery was present on day 7 but resolved by day 45, except for occasional remnants of chronic hemorrhage in the Sham group (Figure 5; Supplementary Material 2, Supplementary Table S2).

Osteonecrosis was rare on day 7, observed at the bone edges of the laminectomy, and absent on day 45. By day 120, osteonecrosis was negligible in the Sham group and absent in the FC and GM groups. The GM group showed a statistically significant reduction from day 7 to day 45, and from day 7 to day 120 (*p* < 0.05), no statistical differences among the groups were found (Figure 5; Supplementary Material 2, Supplementary Table S2).

**Figure 6:**
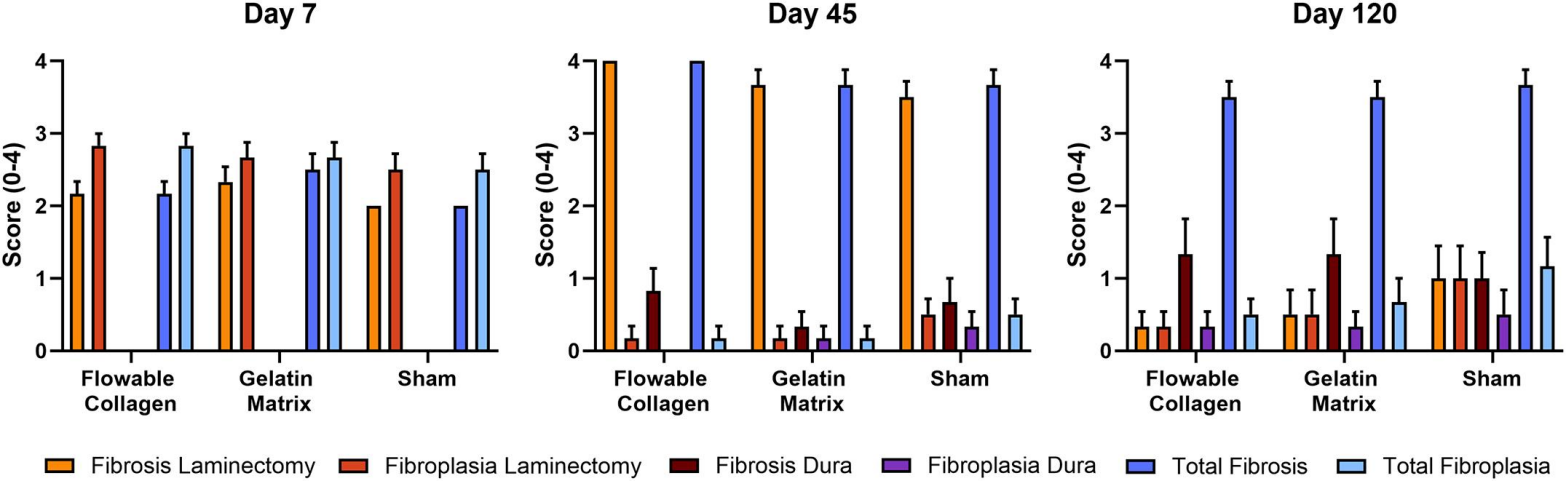
Fibrosis and fibroplasia scores at days 7, 45, and 120 post-treatment. Semiquantitative histological scores showing fibrosis and fibroplasia at days 7, 45, and 120 (mean ± SEM; *n* = 6/group).

Fibrosis was observed in the connective tissue and muscle at the laminectomy site. On day 7, the GM group showed the maximum amount of fibrosis, followed by the FC group and the Sham group (mild to moderate in all groups). By day 45, fibrosis increased in all groups, with the FC group showing the highest levels. No significant differences were found among the groups. Fibrosis was more prominent at the laminectomy site compared with the dura (Figure 6). On day 120, fibrosis decreased in all groups compared with day 45, with minimal dural fibrosis in the Sham group and minimal to mild in the treatment groups. Laminectomy site fibrosis remained higher in the Sham group than in the treatment groups (Figure 6; Supplementary Material 2, Supplementary Table S3).

**Figure 7:**
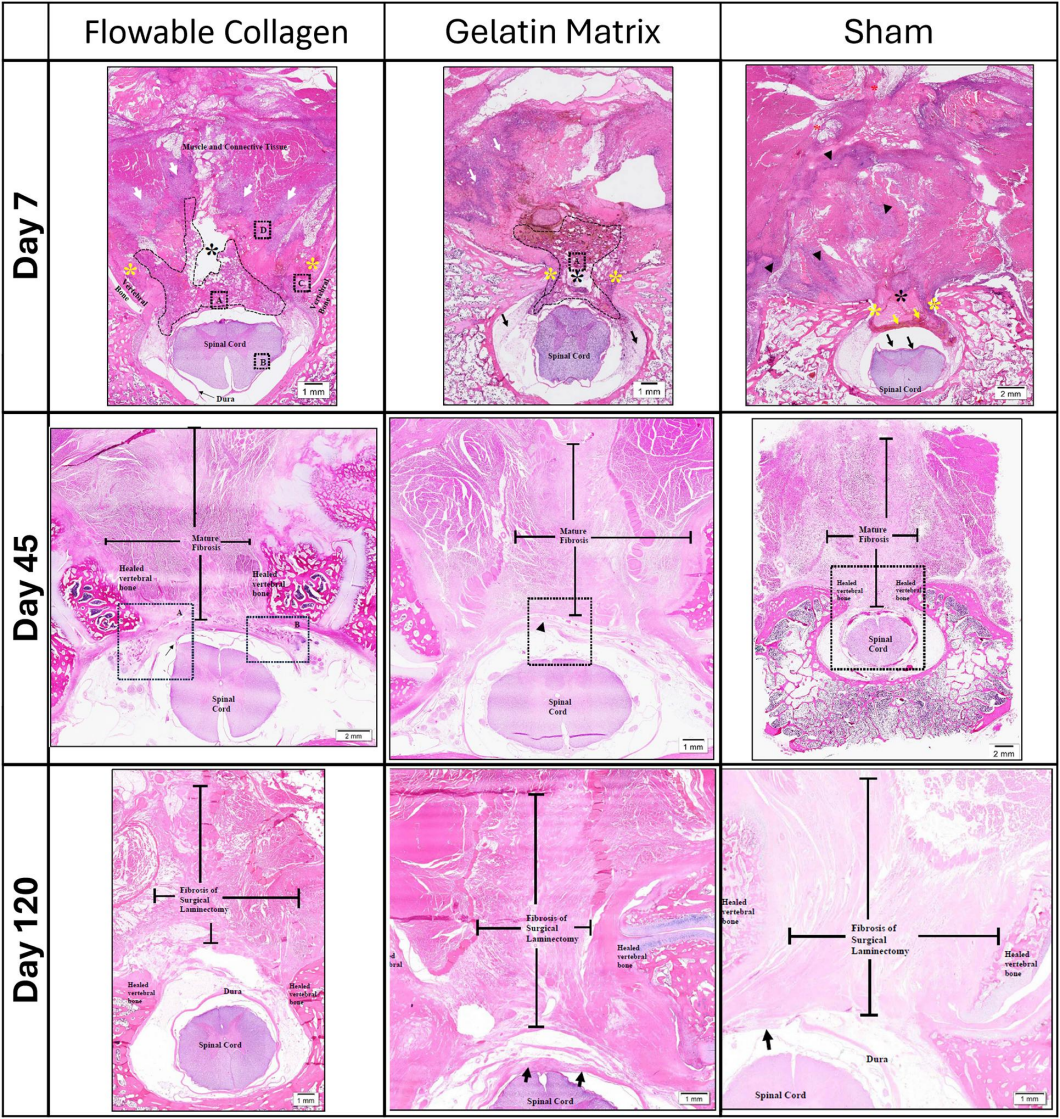
A comparative histology of spinal sites at days 7, 45, and 120 postlaminectomies. H&E-stained sections of L2-L3 spinal sites treated with FC, GM, or Sham. Day 7 FC and GM treatment sites are marked by a dotted area. FC staining shows mild inflammation within the spinal canal and laminectomy site. Inflammation is present on the spinal cord, overlying muscle, and connective tissue. Increased inflammation is indicated by white arrows. In the GM treatment site, there is visible inflammation with fibrin and edema, located in the spinal canal around the spinal cord (indicated by black arrows). The Sham sample has visible partial hematoma (yellow arrows) dorsal to the spinal cord, which is partially compressed (black arrows). Increased inflammation at the laminectomy site is marked with black arrowheads. On day 45 in all treatment sites, laminectomy was replaced with mature fibrosis. FC was slightly detectable (boxed area). The black arrow points to adhesions of the dorsal spinal cord (dorsal horn) to the fibrosing dura. In the GM treatment site, the dura is partially disrupted and thickened with fibrosis (arrowhead). The spinal cord appears normal. For Sham, the entire vertebra section is shown. On day 120, mature fibrosis replacing laminectomy was visible in all sections. There is no visible FC or GM material. The GM samples have a visible thickened dura with fibrosis (black arrows). In the Sham sample, fibrosis of the dura is attached to the overlying connective tissue (black arrow).

Fibroplasia ranged from mild to moderate, with the FC group having the highest amount. By day 45, it was rarely observed in all groups, with the Sham group having the highest amount. At day 120, fibroplasia increased slightly, remaining rare in the FC and GM groups but being minimal to mild in the Sham group. At the dura site, the highest increase of fibroplasia was seen in the Sham group. Similar amounts of fibroplasia were observed in the FC and GM groups on day 120 (Figure 6; Supplementary Material 2, Supplementary Table S3). Representative histological images showing the morphological features underlining quantitative histological scores are presented in Figure 7.

There was no reported macroscopic evidence, at necropsy, indicative of major organ thromboembolism and no macroscopic evidence of hydrocephalus or impaired drainage/flow of the cerebrospinal fluid, and no macroscopic evidence of cerebral thromboembolism. There was no early mortality; all animals survived to their scheduled time point.

## 4 Discussion

Both gelatin and collagen are hemostatic materials with well-studied, favorable biocompatibility and biodegradability. GM is an adjunctive hemostatic agent composed of a bovine-derived gelatin matrix and human thrombin. It aids hemostasis by creating a physical barrier with gelatin granules swelling at the contact with liquid, creating a scaffold and facilitating a stable blood clot formation (10, 16, 19). Thrombin must be added to the gelatin matrix prior to use. As a key enzyme in the coagulation cascade, it activates factors V, VII, and XIII and converts fibrinogen to fibrin during secondary hemostasis, reinforcing clot stability (10, 16). GM swells approximately 10%–20% after application, which could result in compression and necrosis of surrounding tissue (20). It is reabsorbed within 6–8 weeks (16, 21). It has been in use for over 20 years, and multiple studies showed its effectiveness in spinal surgeries (12, 22, 23).

FC is a microfibrillar collagen hemostat (MCH) derived from bovine dermis. It activates both primary and secondary hemostasis. Collagen provides a scaffold for clot formation, directly interacts with receptors (GPVI and *α*_2_*β*_1_) on the platelet surface to trigger their activation, aggregation, and adhesion, and strengthens the clot in primary hemostasis (11, 13, 24). In addition, it binds and activates factor XII, initiating the secondary hemostasis upstream of thrombin, promoting fibrin mesh formation, and stabilizing clot (11, 24). Flowable collagen does not swell after contact with additional fluid and is resorbed within 90 days (13).

Gelatin works by mechanical compression of the tissue to stop bleeding caused by swelling from water absorption and becomes a scaffold for platelet aggregation. Gelatin has weaker biological activity than collagen and does not significantly activate or aggregate platelets (20, 24). When combined with thrombin, its hemostatic activity is enhanced. Thrombin directly participates in the coagulation cascade by converting fibrinogen to fibrin during secondary hemostasis, leading to the formation of a stable clot (8, 19). Collagen, unlike gelatin, is a vital component of the coagulation cascade and is involved in primary and secondary hemostasis as an activator and signaling molecule and provides both structural and biochemical support.

A previously conducted study tested the efficacy and safety of FC in an ovine model of dorsal laminectomy and durotomy in comparison with Avitene™ MCH flour. It demonstrated that flowable formulation allowed for a more uniform coverage with precise application, while not losing any of the hemostatic effects (25). In the current study, both flowable hemostatic devices—FC and GM—showed effective and prompt hemostasis within 3 min in an ovine model of dorsal laminectomy and open durotomy, compared with the Sham treatment group, in which no adjunctive hemostatic agent was used. Flowable collagen, despite not having an active ingredient, performed as well as GM, which includes thrombin.

Microscopic observations confirmed that FC made direct contact with the spinal cord and DRG tissue. However, neurological examinations revealed no differences in adverse findings between animals with hemostatic devices and control animals in the Sham group. This suggests that there was no detrimental effect of the hemostatic agents on the central or peripheral nervous system. Intradural placement of FC and contact with CSF had no impact on the natural sterility of CSF. While *Staph* was identified in 4 CSF samples, the absence of any subsequent infections suggests that it was likely a contaminant from skin surfaces introduced during collection.

Animals treated with both hemostatic devices did not show any discomfort or neurological signs after day 7 for FC and after day 14 for GM. They also had fewer neurological deficits compared with Sham animals whose neurological deficits were more severe and persistent. The FC group showed a decrease in the duration of postoperative side effects compared with the untreated group and is comparable to the GM group.

Previous studies investigated wound healing and inflammatory responses associated with different hemostatic agents (9, 26, 27). Inflammatory response is usually observed around the hemostatic device until complete absorption (26). In the bone defect model, gelatin sponge achieved complete healing, while oxidative regenerative cellulose slowed the rate of repair (26). Studies on the effects of MCH on wound healing reported accelerated wound healing in punch biopsies, possibly due to observed collagen-mediated thrombosis and the promotion of stromal and epithelial cell migration and attachment (27). In a neurosurgical rat model, both GM and Avitene^TM^ MCH showed a tendency to induce granuloma formation (28). While some studies report on increased inflammation associated with the use of MCH in comparison with gelatine-based hemostats, our results suggest that Avitene^TM^ Flowable and GM have similar biocompatibility profiles (26, 29). Both hemostatic device groups had lower histological scores for inflammation than the Sham group on day 120. In addition, based on semiquantitative histopathological scoring, inflammatory cell scores were similar across samples and decreased over time. No excessive fibrosis was reported, and progression was aligned with observed changes in fibroplasia. Although there were no statistical differences among the groups, the highest scores for fibrosis and fibroplasia were observed in the Sham group at the laminectomy site. In contrast, the Sham group had the lowest scores of both fibrosis and fibroplasia at the meninges. These findings contradict those of previous studies that used the rat laminectomy model, which reported the highest fibrosis in animals treated with absorbable collagen, fibrin sealant, and oxidized cellulose. In comparison, treatments with agar and bovine factor proteins, gelatin granules with thrombin, and gelatin paste did not result in fibrosis (30).

Both osteogenesis and osteonecrosis were rare in all groups. Osteonecrosis in both hemostatic device groups dropped to undetectable levels by day 45. Osteogenesis scores decreased over time, but occasionally, osteogenesis/remodeling occurred in all groups on day 120. This may suggest that none of the hemostatic devices affected the bone healing process.

Scores for axonal degeneration were lower on day 45 than on day 7, but by day 120, they had increased to levels similar to those observed on day 7 across all groups. Because axonal degeneration was reported in all groups, it was most likely caused by the associated surgical procedure and not related to the hemostatic devices. Neuronal degeneration of the spinal cord or dorsal root ganglia was not observed. This demonstrates that there was minimal trauma caused by both surgical procedures and use of hemostatic devices. The body's inflammatory and healing responses gradually resolved, which underscores the safety and biocompatibility of hemostatic devices.

In both FC and GM groups, hemostatic material was present on day 7 but significantly decreased to barely detectable levels by day 45. There were no statistically significant differences between the treatment groups, suggesting that both materials were absorbed at similar levels. On day 120, there was one animal in the FC that contained moderate amounts of hemostatic material. Inflammation, necrosis, and mineralization were present at moderate levels, whereas these findings were absent or minimal in other animals in the group. The inflammatory changes were localized to the dura and adjacent connective tissue and did not involve the spinal cord. The underlying cause of more pronounced tissue response in this individual animal cannot be determined based on available data and may reflect interanimal biological variation. This observation was interpreted to be an isolated finding, with no clear evidence indicating an unexpected response related to the use of flowable collagen.

Including the Sham group in the study design allowed differentiation between tissue responses caused by the surgical procedure vs. those attributed to the hemostatic devices. The Sham group exhibited the highest number and longest duration of diverse findings following surgery, indicating that these effects were not device-related. Remnant hemorrhage persisted for the longest period in the Sham group, and fibrosis and fibroplasia were also higher compared with the treatment groups. These observations may suggest poorer healing quality in the Sham group, thereby underscoring the safety profile of both flowable hemostatic devices.

The ovine laminectomy model represents a controlled and reproducible setting for a “worst-case scenario” in humans, given similarities in ovine and human spinal anatomy, physiology, and healing responses. While this model allows for reliable assessment of neurological recovery, it does not fully capture the range of surgical conditions encountered in clinical practice. In addition, the evaluation of both hemostatic devices as an adjunct hemostatic agent was limited to mild to moderate bleeding scenarios and may not reflect their performance in severe or uncontrolled bleeding.

While analyzing the histopathological study results, statistical correction for multiple comparisons was not applied when evaluating differences among groups across three time points, as the study incorporated only a limited number of prespecified comparisons. The analysis was intended to support an exploratory assessment rather than confirmatory hypothesis testing, and the findings should be interpreted descriptively within this context.

Overall, this study demonstrates that flowable collagen is a safe and effective hemostatic agent when applied directly to the spinal cord in an ovine model of dorsal laminectomy and open durotomy. Safety and performance are comparable to the gelatin matrix, a widely used effective hemostatic agent in spinal surgery.

## Data Availability

The original contributions presented in the study are included in the article/Supplementary Material, further inquiries can be directed to the corresponding author.
