# Supplementary material for: Safety and efficacy of flowable microfibrillar collagen hemostat in an ovine model of lumbar laminectomy and open durotomy compared with the gelatin-thrombin hemostatic matrix and control
Source: Front Surg. 2026 Jun 9;13:1760993. doi: 10.3389/fsurg.2026.1760993 (PMC13288811; doi:10.3389/fsurg.2026.1760993)
Supplement: Supplementary file 1 [file Supplementaryfile1.docx]

Supplementary Material 1

# **Evaluation of the Flowable Collagen placement at the acute timepoint**

In addition to the main study (with outcomes assessed on days 7, 45 and 120), an acute experiment (Day 0) was conducted in a smaller group of animals (n=3, Polypay sheep, 34.6-53.8 kg) to confirm appropriate placement of the Flowable Collagen (FC) device in direct contact with the spinal cord at the open durotomy site and at the surgical laminectomy site associated with the spinal canal and/or bone.

The surgical procedure was performed as previously described. Visual assessment of the device swelling, migration and hemostatic efficacy was conducted. Cerebrospinal fluid (CSF) was collected during surgery. Histological evaluation of the laminectomy site, as well as regions proximal/distal to it, was performed.

In the acute group, FC was observed in direct contact with the spinal cord at the open durotomy site and at the surgical laminectomy site associated with the spinal canal and/or bone (Supplementary Figure 1). Supplementary Figure 2 shows representative sections of the spinal cord and dorsal root ganglia (DRG) with the device presence.

As expected at this acute time point, minimal to mild multifocal hemorrhage was observed at the surgical site and within the spinal canal. In one animal a minute amount of FC material was observed lying on the dorsal surface of the spinal cord, accompanied by mild localized axonal degeneration in the underlying dorsal tract. It is unclear if the degeneration was induced as a procedural response or as a response from the device.

The only histological observation was the rare FC material presence associated with the meninges and adjacent to the dorsal root ganglia (DRG). Inflammation and inflammatory cells were not observed in the distal spinal cord or DRG, as expected at this acute time point.


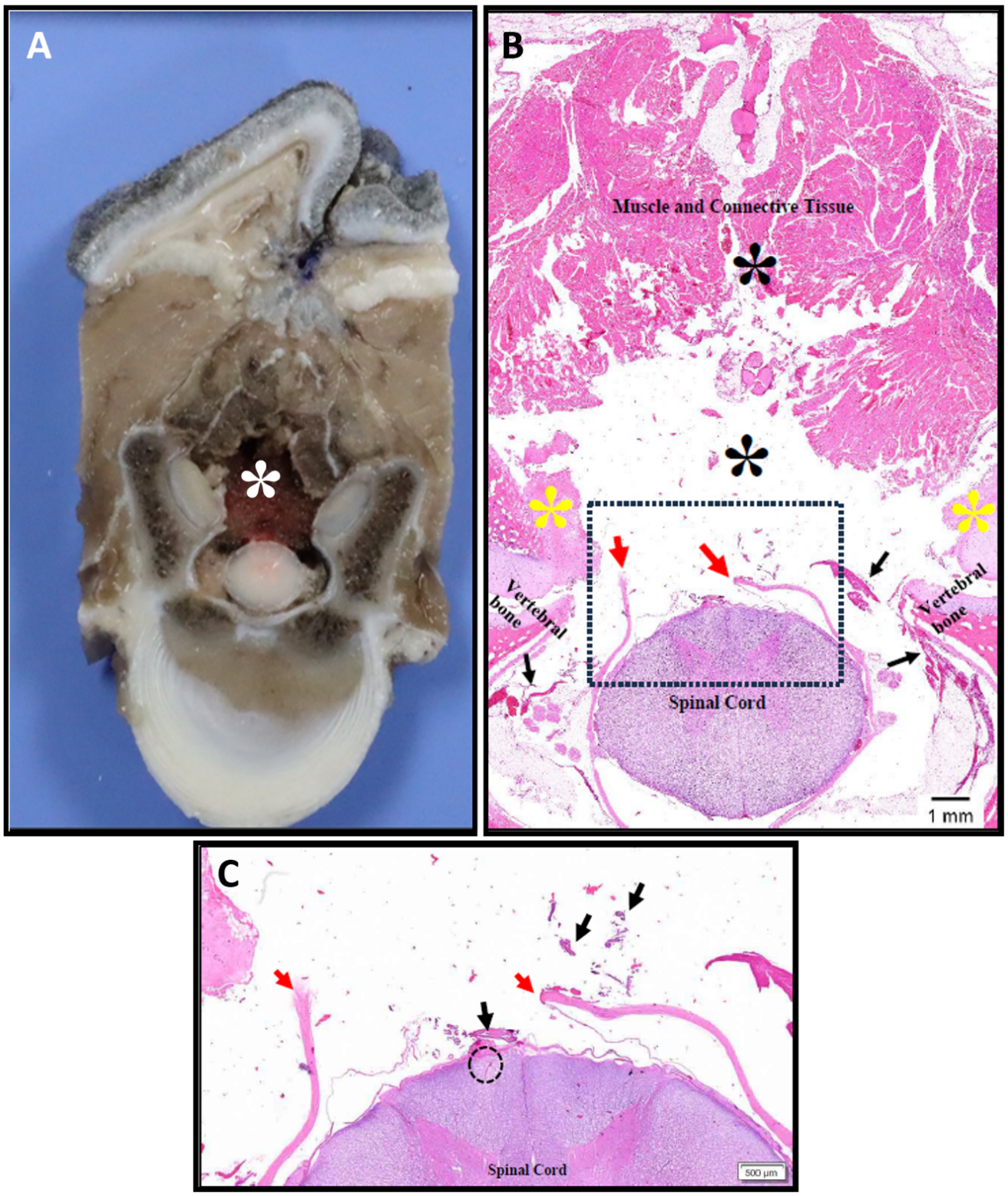


**Supplementary Figure 1.** Macroscopic and microscopic evaluation of FC placement at the acute timepoint.

(A) Macroscopic image showing the treatment site with red material interpreted as FC device mixed with hemorrhage, lying dorsal to and in contact with the spinal cord (white asterisk);

(B) Microscopic image showing an H&E-stained section of FC treatment site (black asterisk), edges of post-laminectomy remaining bone (yellow asterisks), device (dotted area), spinal cord, disrupted dura/durotomy location (red arrows), hemorrhage (black arrows), and overlying muscle and connective tissue;

(C) Higher magnification of dotted box in panel B, showing the device (black arrows), spinal cord with mild focal, axonal degeneration in the dorsal tract (circle), and disrupted dura/durotomy location (red arrows).


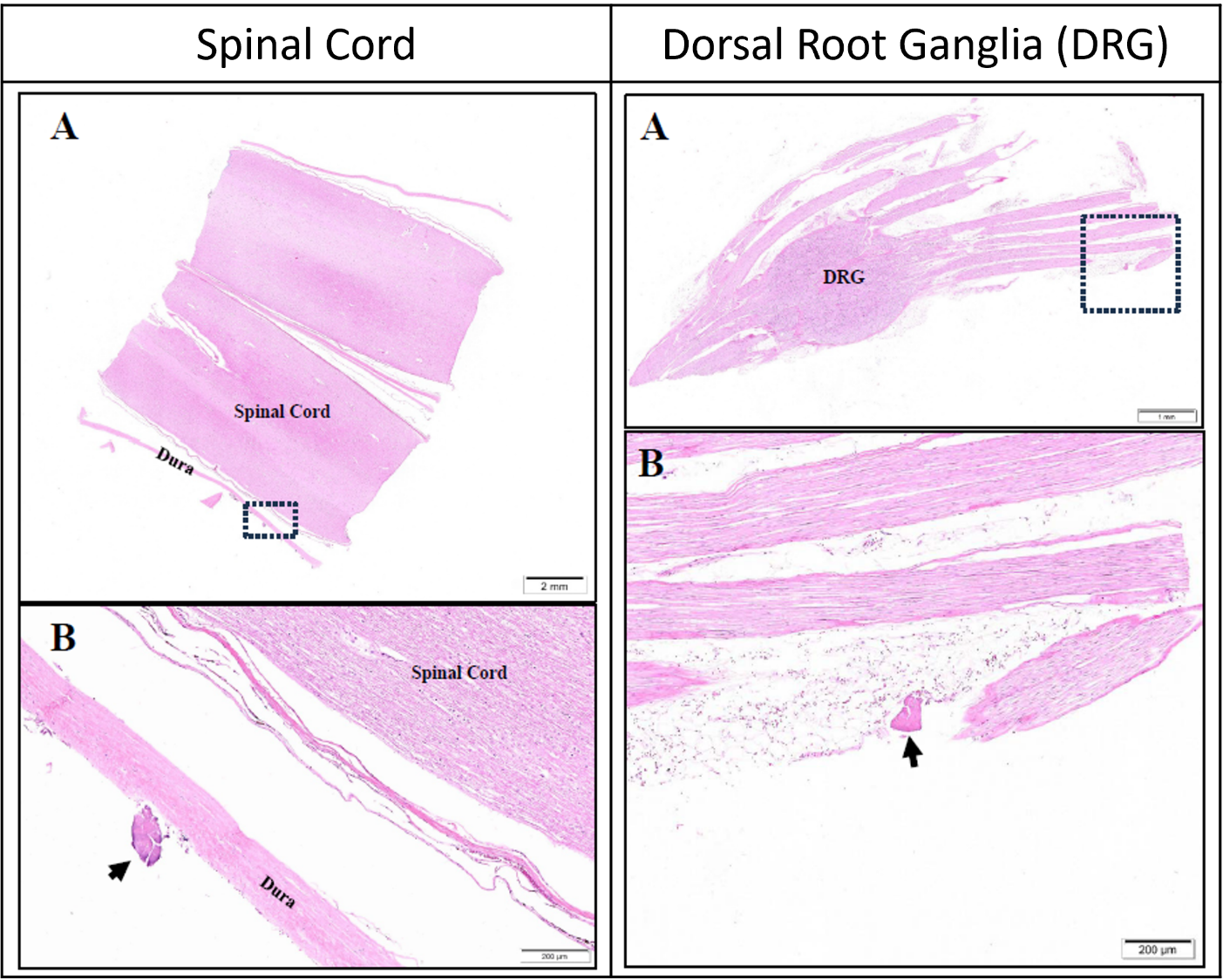


**Supplementary Figure 2.** Microscopic evaluation of spinal cord and DRG following FC placement.

Panels A present low magnification images of H&E-stained section of spinal cord (left) and DRG (right) showing small amounts of FC (dotted area). Panels B show higher magnification of dotted boxes showing rare device material (black arrow).
