# Supplementary material for: Safety and efficacy of flowable microfibrillar collagen hemostat in an ovine model of lumbar laminectomy and open durotomy compared with the gelatin-thrombin hemostatic matrix and control
Source: Front Surg. 2026 Jun 9;13:1760993. doi: 10.3389/fsurg.2026.1760993 (PMC13288811; doi:10.3389/fsurg.2026.1760993)
Supplement: Supplementary file 2 [file Supplementaryfile2.docx]

Supplementary Material 2

**Supplementary Table 1.** Inflammation scores over time post-treatment with Flowable Collagen, Gelatin Matrix, and Sham.

Semi-quantitative histological scores showing inflammation and inflammatory cells at Days 7, 45 and 120 (mean ± SEM; n=6/ group).

|  |  | **Day 7** |  |  |  | **Day 45** |  |  |  | **Day 120** |  |
| --- | --- | --- | --- | --- | --- | --- | --- | --- | --- | --- | --- |
|  | **Flowable Collagen** | **Gelatin Matrix** | **Sham** |  | **Flowable Collagen** | **Gelatin Matrix** | **Sham** |  | **Flowable Collagen** | **Gelatin Matrix** | **Sham** |
| **Inflammation** | 2.50 ± 0.22^§^ | 2.00 ± 0.26^§£^ | 2.00 ± 0.26^§£^ |  | 1.33 ± 0.21 | 1.00 ± 0.00^£^ | 0.83 ± 0.17^£^ |  | 0.83 ± 0.48^§^ | 0.83 ± 0.31^§^ | 1.00 ± 0.00^§^ |
| **Neutrophils** | 1.17 ± 0.17 | 0.83 ± 0.17 | 0.83 ± 0.17 |  | 0.00 ± 0.00 | 0.00 ± 0.00 | 0.00 ± 0.00 |  | 0.17 ± 0.17 | 0.00 ± 0.00 | 0.33 ± 0.21 |
| **Lymphocytes** | 1.67 ± 0.42 | 1.33 ± 0.33 | 1.33 ± 0.42 |  | 1.33 ± 0.21 | 0.50 ± 0.22 | 0.33 ± 0.21 |  | 0.50 ± 0.50 | 0.17 ± 0.17 | 0.33 ± 0.21 |
| **Plasma Cells** | 0.17 ± 0.17 | 0.33 ± 0.21 | 0.50 ± 0.22 |  | 0.50 ± 0.22 | 0.33 ± 0.21 | 0.17 ± 0.17 |  | 0.00 ± 0.00 | 0.17 ± 0.17 | 0.17 ± 0.17 |
| **Macrophages** | 2.17 ± 0.17 | 1.83 ± 0.17 | 2.00 ± 0.26 |  | 1.00 ± 0.00 | 1.00 ± 0.00 | 0.83 ± 0.17 |  | 0.83 ± 0.48 | 0.83 ± 0.31 | 0.83 ± 0.17 |
| **Multinucleated Giant Cells** | 0.67 ± 0.33 | 0.83 ± 0.31 | 0.50 ± 0.34 |  | 0.83 ± 0.17 | 0.50 ± 0.22 | 0.33 ± 0.21 |  | 0.33 ± 0.33 | 0.00 ± 0.00 | 0.00 ± 0.00 |
| **Mast Cells** | 0.00 ± 0.00 | 0.00 ± 0.00 | 0.00 ± 0.00 |  | 0.00 ± 0.00 | 0.17 ± 0.17 | 0.00 ± 0.00 |  | 0.00 ± 0.00 | 0.00 ± 0.00 | 0.00 ± 0.00 |

* - statistical significance between groups; § - statistical significance between scores on Day 7 and Day 120; £ - statistical significance between scores on Day 7 and Day 45

**
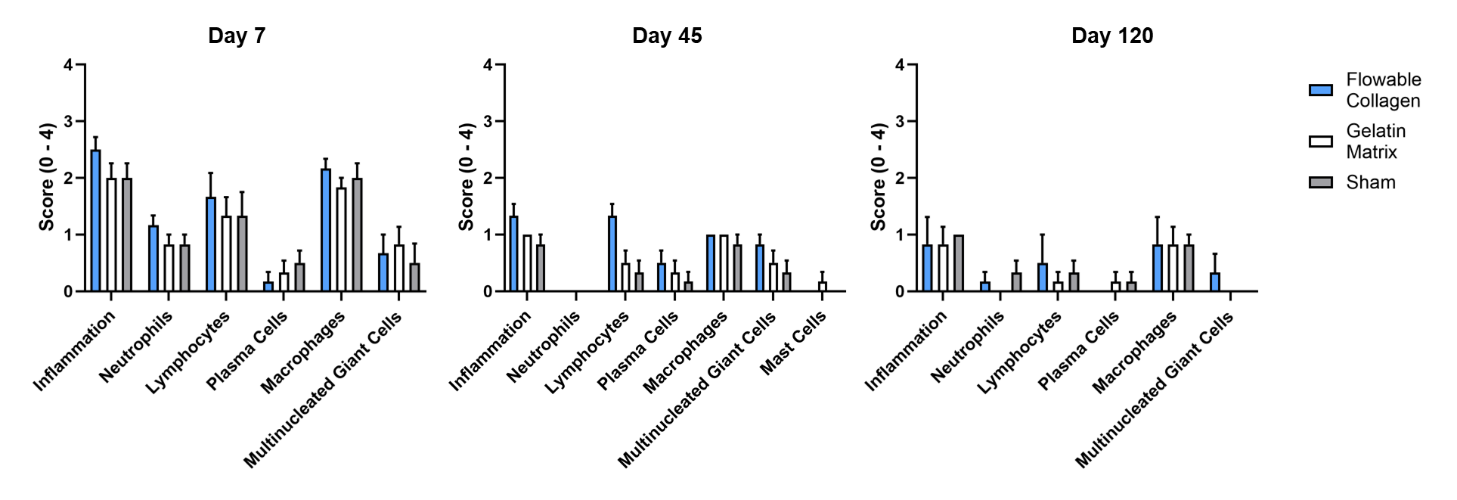
**

**Supplementary Figure 1.** Inflammation and inflammatory cell scores by parameter, treatment group and timepoint for Flowable Collagen, Gelatin Matrix, and Sham.

Semi-quantitative histological scores showing inflammation and inflammatory cells at Days 7, Day 45, and Day 120 (mean ± SEM; n=6/ group).

**Supplementary Table 2.** General histological observations at Days 7, 45 and 120 post-treatment.

Semi-quantitative histological scores of general tissue observations at Days 7, 45 and 120 (mean ± SEM; n=6/ group).

|  |  | **Day 7** |  |  |  | **Day 45** |  |  |  | **Day 120** |  |
| --- | --- | --- | --- | --- | --- | --- | --- | --- | --- | --- | --- |
|  | **Flowable Collagen** | **Gelatin Matrix** | **Sham** |  | **Flowable Collagen** | **Gelatin Matrix** | **Sham** |  | **Flowable Collagen** | **Gelatin Matrix** | **Sham** |
| **Axonal Degeneration** | 0.50 ± 0.34 | 0.50 ± 0.22 | 0.5 ± 0.22 |  | 0.00 ± 0.00 | 0.17 ± 0.17 | 0.17 ± 0.17 |  | 0.50 ± 0.22 | 0.33 ± 0.21 | 0.67 ± 0.21 |
| **Presence of Device Material** | 3.00 ± 0.00^§£^ | 3.00 ± 0.00^§£^ | 0.00 ± 0.00* |  | 0.67 ± 0.33^£^ | 0.33 ± 0.21^£^ | 0.00 ± 0.00 |  | 0.50 ± 0.50^§^ | 0.00 ± 0.00^§^ | 0.00 ± 0.00 |
| **Osteonecrosis** | 0.67 ± 0.21 | 0.83 ± 0.17^§£^ | 0.83 ± 0.17 |  | 0.00 ± 0.00 | 0.00 ± 0.00^£^ | 0.00 ± 0.00 |  | 0.00 ± 0.00 | 0.00 ± 0.00^§^ | 0.17 ± 0.17 |
| **Osteogenesis/**  **Remodeling** | 0.50 ± 0.22 | 0.50 ± 0.22 | 0.50 ± 0.22 |  | 0.83 ± 0.17 | 0.50 ± 0.22 | 0.50 ± 0.22 |  | 0.17 ± 0.17 | 0.17 ± 0.17 | 1.00 ± 0.36 |

* - statistical significance between groups; § - statistical significance between scores on Day 7 and Day 120; £ - statistical significance between scores on Day 7 and Day 45


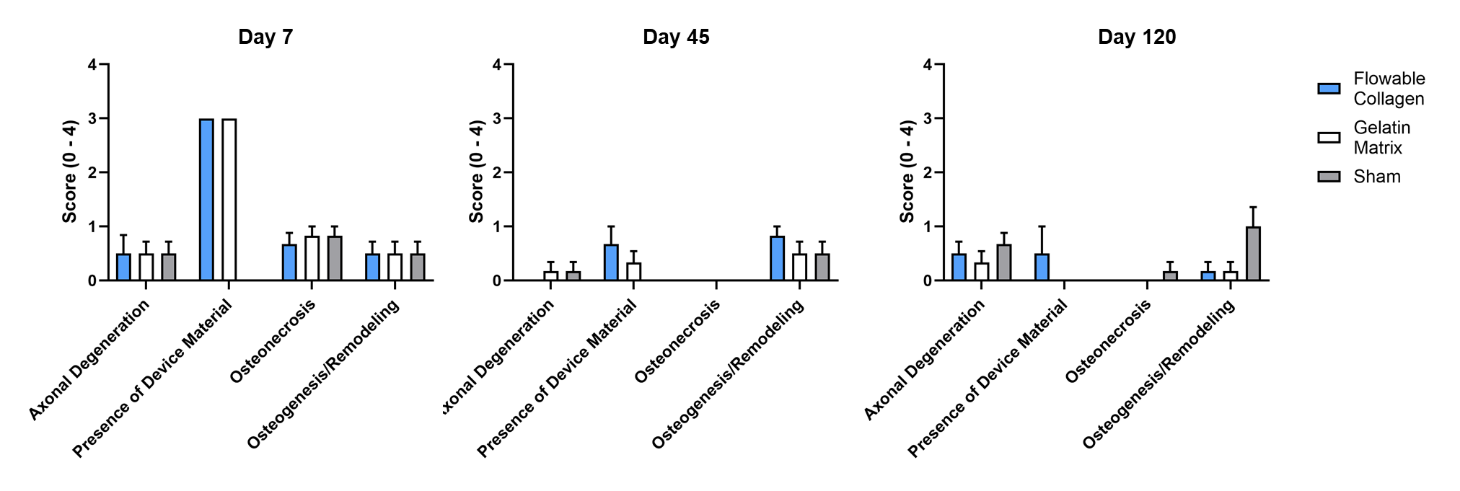


**Supplementary Figure 2.** General histological observations by parameter, treatment group and timepoint for Flowable Collagen, Gelatin Matrix, and Sham.

Semi-quantitative histological scores showing inflammation and inflammatory cells at Days 7, Day 45, and Day 120 (mean ± SEM; n=6/ group).

**Supplementary Table 3.** Fibrosis and fibroplasia scores at Days 7, 45, and 120.

Semi-quantitative histological scores showing fibrosis and fibroplasia at Days 7, 45 and 120 (mean ± SEM; n=6/ group).

|  |  | **Day 7** |  |  |  | **Day 45** |  |  |  | **Day 120** |  |
| --- | --- | --- | --- | --- | --- | --- | --- | --- | --- | --- | --- |
|  | **Flowable Collagen** | **Gelatin Matrix** | **Sham** |  | **Flowable Collagen** | **Gelatin Matrix** | **Sham** |  | **Flowable Collagen** | **Gelatin Matrix** | **Sham** |
| **Total Fibrosis** | 2.17 ± 0.17 | 2.50 ± 0.22 | 2.00 ± 0.00 |  | 4.00 ± 0.00 | 3.67 ± 0.21 | 3.67 ± 0.21 |  | 3.50 ± 0.22 | 3.50 ± 0.22 | 3.67 ± 0.21 |
| **Total Fibroplasia** | 2.83 ± 0.17 | 2.67 ± 0.21 | 2.5 ± 0.22 |  | 0.17 ± 0.17 | 0.17 ± 0.17 | 0.5 ± 0.22 |  | 0.50 ± 0.22 | 0.67 ± 0.33 | 1.17 ± 0.40 |
| **Fibrosis Laminectomy** | 2.17 ± 0.17 | 2.33 ± 0.21 | 2.00 ± 0.00 |  | 4.00 ± 0.00 | 3.67 ± 0.21 | 3.5 ± 0.22 |  | 0.33 ± 0.21 | 0.50 ± 0.34 | 1.00 ± 0.45 |
| **Fibroplasia Laminectomy** | 2.83 ± 0.17 | 2.67 ± 0.21 | 2.5 ± 0.22 |  | 0.17 ± 0.17 | 0.17 ± 0.17 | 0.5 ± 0.22 |  | 0.33 ± 0.21 | 0.50 ± 0.34 | 1.00 ± 0.45 |
| **Fibrosis Dura** | 0.00 ± 0.00 | 0.00 ± 0.00 | 0.00 ± 0.00 |  | 0.83 ± 0.31 | 0.33 ± 0.21 | 0.67 ± 0.33 |  | 1.33 ± 0.49 | 1.33 ± 0.49 | 1.00 ± 0.36 |
| **Fibroplasia Dura** | 0.00 ± 0.00 | 0.00 ± 0.00 | 0.00 ± 0.00 |  | 0.00 ± 0.00 | 0.17 ± 0.17 | 0.33 ± 0.21 |  | 0.33 ± 0.21 | 0.33 ± 0.21 | 0.50 ± 0.34 |

* - statistical significance between groups; § - statistical significance between scores on Day 7 and Day 120; £ - statistical significance between scores on Day 7 and Day 45


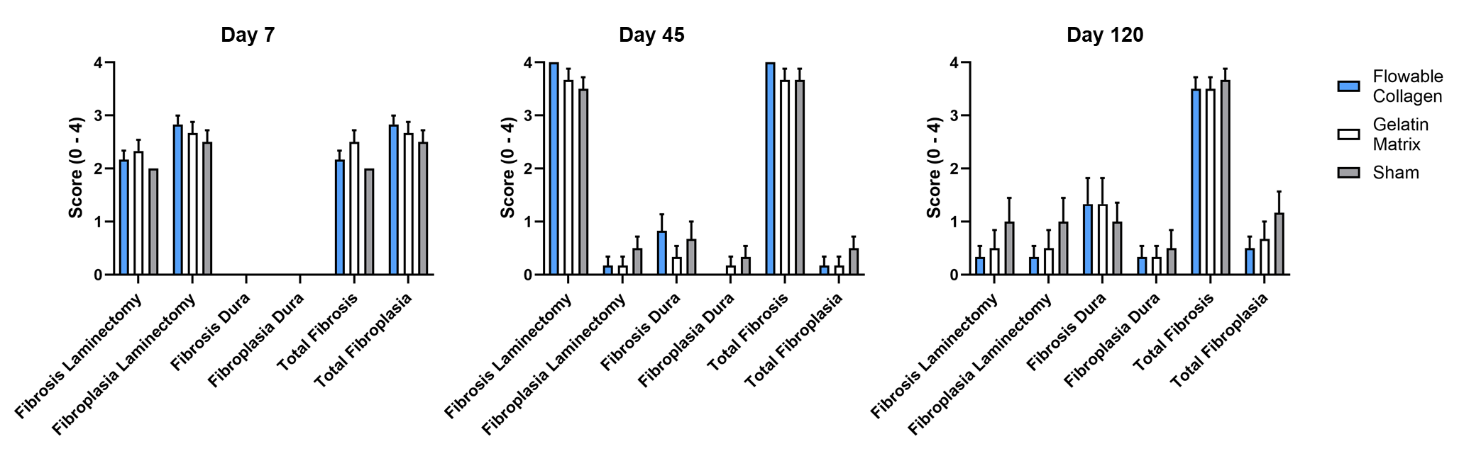


**Supplementary Figure 3.** Fibrosis and fibroplasia scores by parameter, treatment group and timepoint for Flowable Collagen, Gelatin Matrix, and Sham.

Semi-quantitative histological scores showing inflammation and inflammatory cells at Days 7, Day 45, and Day 120 (mean ± SEM; n=6/ group).
